# How Attractive Is the Girl Next Door? An Assessment of Spatial Mate Acquisition and Paternity in the Solitary Cape Dune Mole-Rat, *Bathyergus suillus*

**DOI:** 10.1371/journal.pone.0039866

**Published:** 2012-06-29

**Authors:** Timothy C. Bray, Paulette Bloomer, M. Justin O’Riain, Nigel C. Bennett

**Affiliations:** 1 Department of Zoology and Entomology, Mammal Research Institute, University of Pretoria, Pretoria, South Africa; 2 Molecular Ecology and Evolution Programme, Department of Genetics, University of Pretoria, Pretoria, South Africa; 3 Department of Zoology, University of Cape Town, Cape Town, South Africa; Ecole Normale Supérieure de Lyon, France

## Abstract

Behavioural observations of reproduction and mate choice in wild fossorial rodents are extremely limited and consequently indirect methods are typically used to infer mating strategies. We use a combination of morphological, reproductive, spatial, and genetic data to investigate the reproductive strategy of a solitary endemic species, the Cape dune mole-rat *Bathyergus suillus.* These data provide the first account on the population dynamics of this species. Marked sexual dimorphism was apparent with males being both significantly larger and heavier than females. Of all females sampled 36% had previously reproduced and 12% were pregnant at the time of capture. Post-partum sex ratio was found to be significantly skewed in favour of females. The paternity of fifteen litters (n = 37) was calculated, with sires assigned to progeny using both categorical and full probability methods, and including a distance function. The maximum distance between progeny and a putative sire was determined as 2149 m with males moving between sub-populations. We suggest that above-ground movement should not be ignored in the consideration of mate acquisition behaviour of subterranean mammals. Estimated levels of multiple paternity were shown to be potentially as high as 26%, as determined using sibship and sire assignment methods. Such high levels of multiple paternity have not been found in other solitary mole-rat species. The data therefore suggest polyandry with no evidence as yet for polygyny.

## Introduction

Mammals display a wide range and flexibility of behavioural and social interactions (e.g. [1]), which is well exemplified in their breeding systems. The most common mammalian mating system is polygyny [2], [3] where male mate acquisition ranges from opportunistic approaches [4], to the competitive attainment of a social or spatial position that confers improved access to females (e.g. [5]). In some species spatial proximity to potential mates reduces aggression and facilitates mating opportunities [6], [7]. Attenuated aggression through proximity may be particularly important for subterranean species which experience high energetic costs associated with extending burrow systems in order to locate potential mates.These energetic restrictions may be relaxed by the adoption of alternate strategies such as surface movement [8] or the use of adjoining burrow systems. Although males commonly attempt to monopolise females to improve reproductive success, they seldom successfully exclude all competing suitors [9]. Solitary multiparous mammalian species that do not form pair-bonds may thus be expected to have high levels of multiple paternity (e.g. [10]).

The inference of mating system from morphological information is well established (e.g. [11]) but can be problematic, particularly where mixed mating systems are found [12]. Male reproductive success is often highly variable [13] due in part to interplay between multiple mating strategies which vary both in frequency and temporally [14]. The apparent adoption of one mating system often belies complexities such as extra pair copulations [15]. Unravelling the mating dynamics in cryptic systems such as subterranean organisms is reliant on comprehensive investigations incorporating genetic, spatial, and morphological information (e.g. [16]). While familial relationships can be determined directly through genotypes, population level dynamics can be inferred from population characteristics such as reproductive skew and associated discrepancies in sex ratio [17].

The family Bathyergidae is a group of subterranean rodents found throughout sub-Saharan Africa [18]. Initially eclipsed by the discovery of eusociality in the social genera (e.g. [19]) solitary mole-rat species have only recently begun to receive research interest [8], [20], [21]. The Cape dune mole-rat, *Bathyergus suillus* is one of two solitary members of the genus *Bathyergus* [22] which is largely restricted to mesic regions [23] characterised by a sandy substrate, in the South African Western Cape [18]. Little is known about the mating system in *B. suillus* and based on morphology conflicting suggestions of both polyandry and polygny have been proposed [24], [25]. Assumptions about mating systems have been cautioned in the absence of genetic data [26]. Here we use a combination of demographic, morphological, spatial, reproductive, and genetic data on a large free-ranging population of *B. suillus* to shed light on the mating system of this endemic, fossorial rodent.

No demographic information is available for the Cape dune mole-rat and no population genetic information has been presented previously. Primarily we take a genetic approach to characterise the mating system in this subterranean rodent. We attempt to determine the degree to which a subterranean ecology limits the distance over which a male may sire progeny. Additionally, we use morphological and sex ratio information to give further insight into the mating dynamics of this species.

## Methods

### Sampling

**Figure 1:**
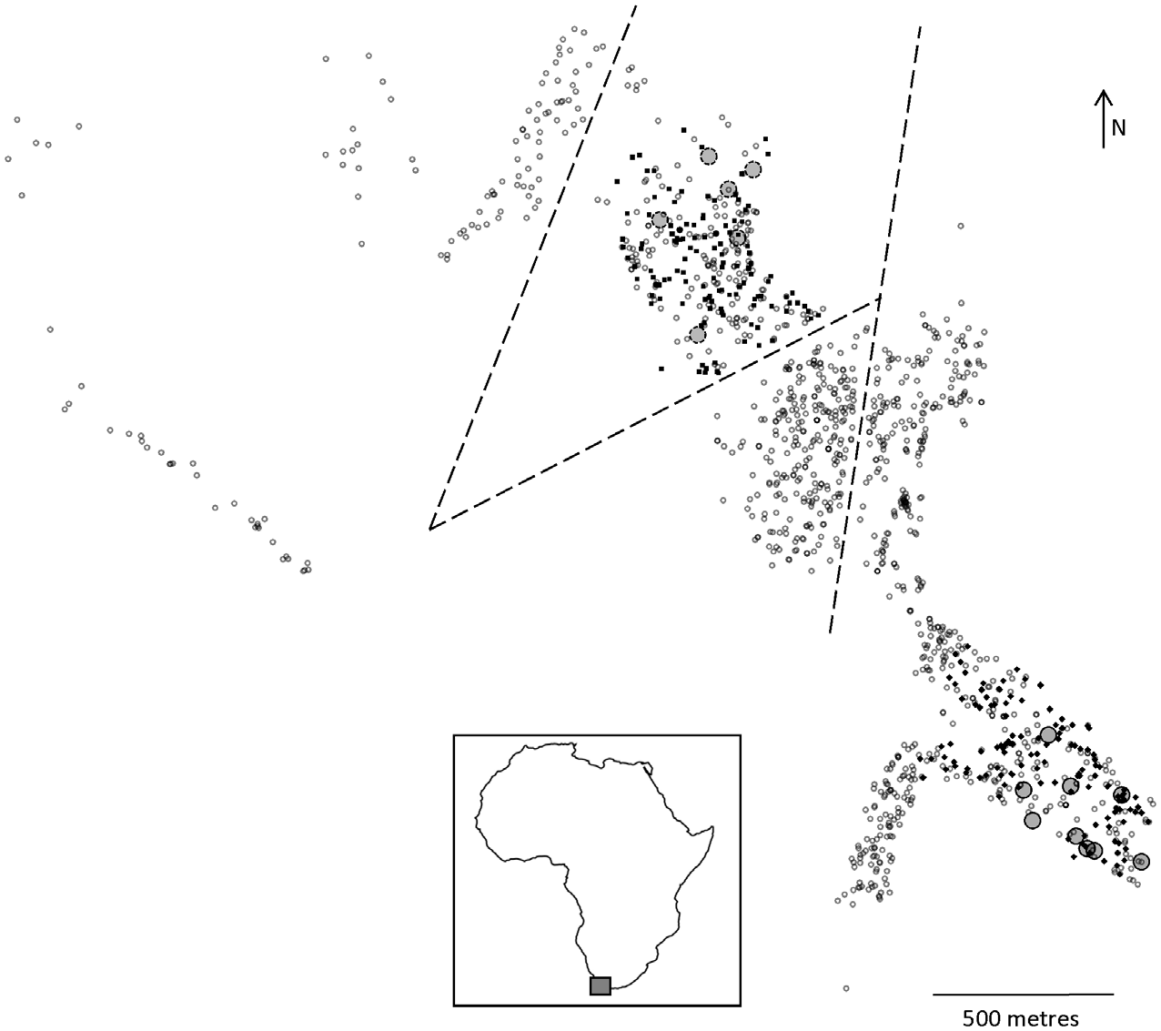
Map of *B. suillus* individuals sampled. The south-eastern study area is Region 1 showing study females (filled grey circles, solid lines) and potential sires (black diamonds). Region 2 is the northern study area showing study females (filled grey circles, dashed lines) and potential sires (black squares). Tarred runways and access roads representing potential barriers to subterranean movement are represented by dashed lines. Sampled individuals not genotyped are also shown (small unfilled circles).

Morphological, spatial and reproductive data were obtained for 1350 *B. suillus* individuals that were captured and humanely euthanised as part of an eradication program run by the Airports Company of South Africa at Cape Town International Airport (see [27] for details). All procedures conformed to the guidelines of the American Society of Mammalogists [28]. The collection and processing of all biological material used in this study was approved by the University of Cape Town Animal Ethics Committee (AEC#:2003/V&/JOR). Muscle tissue samples were taken and stored in ethanol. Included in the sampling were 21 gravid females. Spatial and genetic data were analysed for a subset of the total sample from two different regions within the airport grounds that were separated by below ground barriers (runways) that prevented any subsurface movement (Fig. 1). We used these two regions to investigate the relationship between paternity and distance from sampled pregnant females. Region 1 contained nine gravid females (2-3 progeny per litter, n = 23 total) and a total of 143 potential sires (all males within 400 m of a sampled female). Region 2 contained six gravid females (1-4 progeny per litter, n = 14 total) with 152 potential sires (Fig. 1). DNA samples were collected for all females with progeny and their litters, as well as 132 males from region 1 and 139 males from region 2. An additional nine ‘transient’ individuals caught above-ground and hence with no location information were treated as potential sires for both regions.

### Genotyping

Genotypes were generated from 19 microsatellite markers developed for *B. suillus* and related species; BS01, BS07 ([29], GenBank accession numbers; HQ186238-45); CH1, CH3, DM1, DM5, DM7 ([30], GenBank accession numbers; AF380165-75); Gcap02, Bsuil06, Bsuil04, Chott03, Cmech04, Bsuil02, Gcap07, Bsuil01, Chott05, Cmech03, Gcap10, Cmech09 [31]. These markers were applied in four multiplexes using the Qiagen Multiplex kit with standard conditions (45 cycles, 60°C annealing temperature). Heterozygosity scores and linkage disequilibrium for all locus pairs were calculated in GENETIX4.05 [32], as well as population-level summary statistics. All loci were tested for conformation to Hardy-Weinberg Equilibrium and for the presence of null alleles using GENEPOPv4.0.10 [33]. A migrant analysis was performed using GENECLASS2 [34]. Genotyping error rate across the dataset was also quantified by repetition of ∼10% of the individuals across all loci.

### Capture Location and Spatial Analyses

GPS points were taken at the point of capture for all individuals sampled, and coordinates were mapped using ARCGIS 10 [35]. For the purposes of this study the capture coordinates were applied as if they were the centre of the home burrow system. We calculated mean distances between all males and females in both regions 1 and 2. We used an individual-based ‘isolation by distance’ approach across just the males, under an area model using GENEPOPv4.0.10 [33] to investigate the relationship between spatial patterns and genetic variation in both regions.

### Parentage Analysis

In accordance with previous recommendations [36] two statistical approaches were applied to assign parentage based on the genetic data:

#### (i) Categorical allocation

The most likely sires are calculated from the non-excluded putative sires within a likelihood framework. Calculations are based on the probability of the parental genotype producing the alleles required to construct the genotype of the progeny. An advantage of this approach is that it allows the accommodation of scoring errors and mutations. Sire determinations were made using the CERVUS [37] algorithm (within the R package MASTERBAYES; [38] for paternity assignment to give the single most likely sire for each of the progeny.

#### (ii) Full probability

Here the specified model allows inclusion of other explanatory variables in addition to the genotype information according to the algorithm of MASTERBAYES [38]. Spatial information was included in the sire assignment to compare against the basic categorical allocation. A further advantage of this approach is the inclusion of a measure of uncertainty.So as to include the transient males into the distance analysis they were given the same geographic location outside the bounds of the region to reflect their unknown origin.

For both of these approaches analyses were performed through the MASTERBAYES [38] application in R under two scenarios: Each statistical approach was modelled for the situation assuming all sires are present (Allsire) and adopting the known rates of sire absenteeism (USsire). Absent males numbered 11 in region 1 and 13 in region 2. Sire assignment was determined separately for each region and then repeated using all sampled males from both regions. It is known that genotyping error of as low as 1% can have a strong effect on paternity assignment and exclusion [39]. We considered genotyping error rates for the dataset in the analysis employing values of 0.1, 1, and 5% for comparison. Maximum likelihood initial parameterisations are used as default with parameters being estimated by the model. The maximum likelihood pedigree was fixed with regard to the inclusion of known mothers. The two approaches employed here differ in their treatment of genotyping error; The CERVUS approach assumes the second allele in an erroneous genotype is also mis-scored, and that the true genotype is a reflection of genotype frequency in the population [40]. The MASTERBAYES error is calculated as equally likely across all alleles, with a second error term for the likelihood of the dropout of alleles from a heterozygote genotype [41].

### Relatedness and Multiple Paternity

Relatedness was measured and the distribution of relatedness values described within each region using the ML-RELATE application [42]. This application calculates the maximum likelihood relationship between individuals based on genetic data. Relatedness was calculated between littermates to further investigate the incidence of multiple paternity across all 19 litters with two or more siblings. The analysis was performed according to the ‘Matrix’ setting for comparison of multiple individuals. Total allele numbers at each locus in the genotypes of those nine litters containing three or more progeny were also compiled as a third means by which to estimate multiple paternity.

### Morphology and Sex

Total body length, mass, sex, and reproductive status measurements were recorded (sensu [27]) for 1350 individuals. Means for male and female length and body mass were compared using a T- test under the null hypothesis of no difference between distributions. The shape of the frequency distribution was modelled for both length and mass in each gender using the MCLUST [43] application. A sub-sample of females was investigated for uterine scarring and pregnancy (n = 518). All sex ratios were evaluated for significance using a Chi-Squared test (null hypothesis no deviation from 1∶1). Molecular sexing was used to examine sex ratios of foetuses, using the DBY markers and protocols developed for *Heterocephalus glaber* [44]. DBY amplification was less efficient in *B. suillus* than *H. glaber* and thermocycling conditions were altered to 45 cycles at an annealing temperature of 45°C. As absence of DBY amplification indicates a female, individual DNA was confirmed using mitochondrial DNA 16S amplification prior to a minimum of three independent DBY amplifications with positive controls.

## Results

### Genotyping

Deviations from Hardy-Weinberg Equilibrium and estimates of null alleles are shown in Appendix S1. Locus D1 has a highly significant heterozygote deficiency (P<0.001) and an estimated null allele frequency of 0.32, above arecommended 0.2 upper bound [45]. For these reasons locus D1 was excluded from further analyses. The test for linkage disequilibrium showed no significant association between alleles across loci (all p>0.05). Population genetic summary statistics are given in Table 1. There was a low but highly significant genetic differentiation (F_ST_ = 0.018, P<0.001) between the two regions (using ten representative members of each sex from each region). Detection of first generation migrants suggested 20 migrants each way between the two regions at the most strict assignment level (P = 0.001). Genotyping error was calculated as being 0.018%.

**Table 1:** Genetic summary statistics for *B.suillus* individuals for region 1, region 2, and total adults (including both regions and transient males).

	H_E_	H_O_	A
Region 1	0.69	0.64	8.3
Region 2	0.67	0.62	8.1
Total	0.69	0.63	9.1

Expected and observed heterozygosity (H_E_, H_O_), and number of alleles per locus (A).

### Geographical Information

The mean distances within and between the sexes in regions 1 and 2 are provided in Table 2. A Mantel test of genetic distance against geographic distance indicated no statistically significant correlation within regions (10000 permutations; P = 0.001; number of pairs = 10153, 11476 for region 1 and 2 respectively), suggesting no pattern of natal philopatry in males across this site.

**Table 2:** Mean regional separation distances in metres between members of each gender (standard deviations in parentheses).

	Region 1		Region 2	
	Male	Female	Male	Female
Male	326(198)		276(123)	
Female	336(199)	343(205)	249(134)	277(155)

### Parentage Analysis

Sire assignment to progeny was seen to be affected by statistical approach and whether all males were considered sampled (Appendix S2). Including distance as a variable in the full probability approach did not change sire assignments. Subsequent to initial analyses and in consideration of the error rate of 0.018% determined for the dataset, assigned sires were considered only if two or fewer mismatches were present (total alleles in each assignment taking into account dam: 3*(18*2)*0.018 = 1.9 mismatches). Due to the low level of within region sire assignment according to these criteria, results presented are for the inclusion of all sampled males from both regions in each analysis. When all males were assumed sampled (ALLsire) 10/23 progeny were assigned sires by one or both methods in region 1, and 9/14 in region 2. Methods agreed on five assignments in region 1, and three in region 2. For the remaining assignments only one method assigned a sire in each case apart from two instances in region 2 where the two methods assigned different sires. When the assumption of all males sampled was relaxed (USsire) two assignments were made in region 1 (both methods in agreement; all probability>0.95) with the full probability method assigning a further three at lower confidence (probability<0.6). In region 2 a single USsire assignment was made in region 2 by the categorical approach only (probability = 0.23). Alteration of error parameter did not affect sire assignments but decreasing error increased each assignment probability by an average 0.04 in region 1 and 0.03 in region 2 in the categorical approach only.

### Calculating Mate Acquisition Distances

We inferred mate acquisition distances in two ways from these data: Level of sire assignment (i.e. proportion of unsampled sires), and from direct measurements for sire-progeny assignments (Appendix S2). The drop in assignment rate when the possibility of unsampled males is introduced suggests that a potentially high proportion of unsampled males have travelled further than the sampling radius to sire offspring. Sampling radius is a minimum of 400 m for region 1 and ∼200 m for region 2. The greatest sire-progeny distance calculated for the ALLsire model where both approaches gave the same sire was 2149 m. The greatest consensus sire distance in the more conservative USsire model was 34 m. Distances above 1000 m represent movement between regions.

### Determination of Multiple Paternity and Relatedness

Two of nine litters showed evidence of multiple paternity at a single locus (three or more non-maternal alleles present), a further two litters showed evidence of multiple paternity over two loci. Using the statistical approach of the MLrelate algorithm for determining sibship, five of 19 litters were found to contain half-siblings. We also inferred multiple paternity from those sire assignments where different males were assigned to a single litter with a probability of assignment of at least 0.8; Sire assignments across methods for the 15 litters suggest two litters contained two siblings attributed to the same sire, one attributed siblings to two different sires, and nine did not have more than one sire assignment for comparison. Relatedness estimates for region 1 and 2 were 0.05 and 0.06 (variance 0-0.67 and 0-0.83; number of pairs 13366 and 12561 respectively). Inter-litter relatedness was low within each region (0.025, 0.028 respectively) and both were significantly different from the regional relatedness (T-test;region 1, df = 10078, P<0.0001; region 2, df = 10516, P = 0.02).

### Morphometrics and Sexing

**Figure 2:**
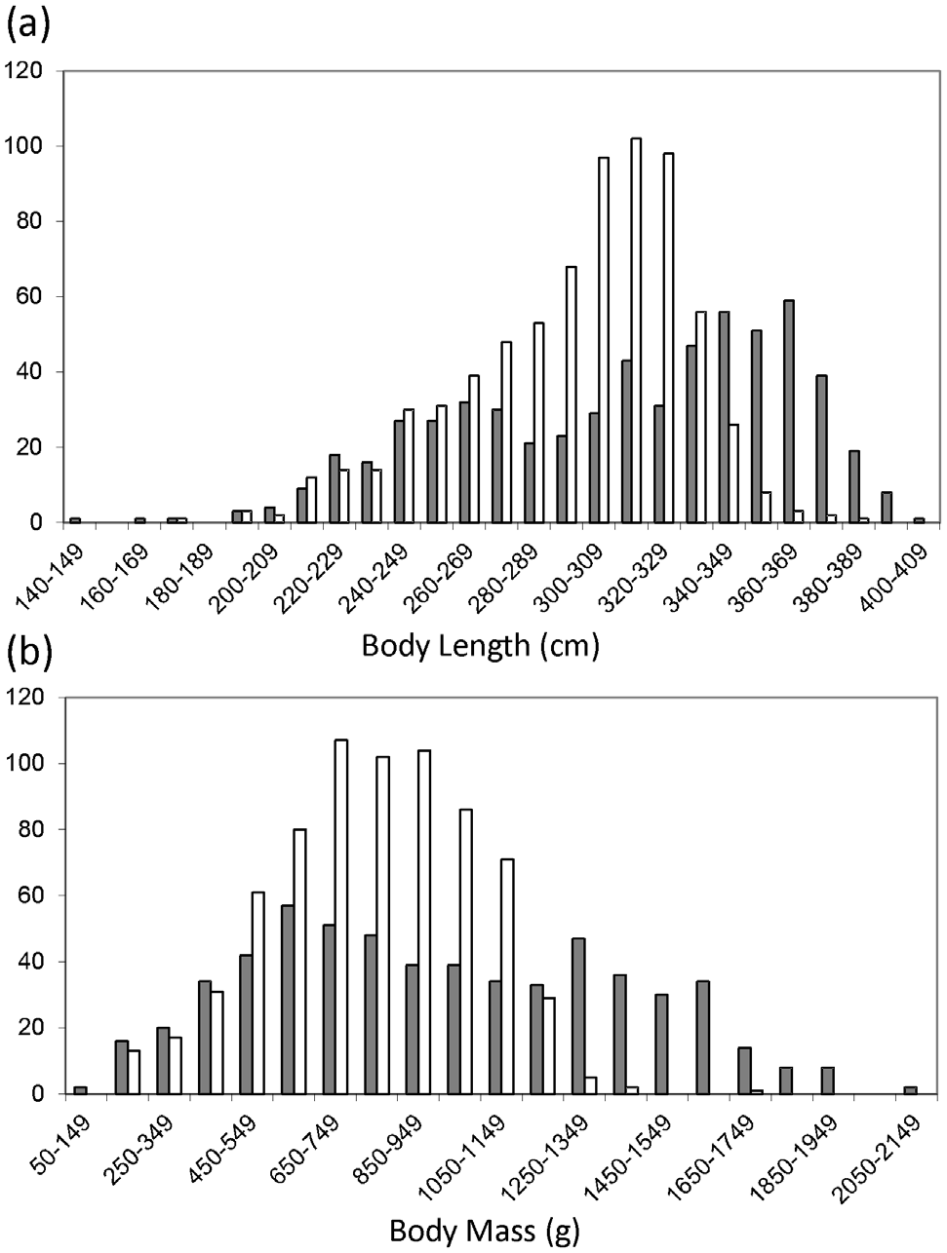
Distribution of morphological measurements by sex. Body length (a) and body mass (b) distributions for sampled male (grey) and female (white) Cape dune mole-rats, *B. suillus*.

Overall mean values showed that males were significantly longer (mean = 311 mm, T-test; df = 938, P>0.0001) and heavier (mean = 955 g, T-test; df = 935, P>0.0001) than females (788 g, SD = 243; 294 mm, SD = 33, respectively) (Fig. 2a and 2b). Frequency distributions of body length and body mass reveal a unimodal distribution for females. Both length and body mass of males were best explained by models with two components (Appendix S3; length = 275, 350 mm, mass = 650,1400 g). Of the 518 females sampled 36% had uterine scarring, while 12% were pregnant on capture. The adult sex ratio for the study site was significantly female biased with a ratio of 0.84∶1 (df 1, X^2^ 9.8, P>0.001, n = 1305) whilst the sex ratio of unborn foetuses from 21 adult females was 0.76∶1 (df 1, X^2^ 0.96, NS, n = 51).

## Discussion

In this study, genetic analyses have provided evidence for multiple paternity in a fossorial, solitary rodent with males appearing to travel considerable distances to acquire mates. This mirrors the findings in another solitary rodent, *Heliophobius argenteocinereus* [8]. These data suggest a mating system with males competing with one another for access to females and the latter accepting multiple partners.

### Population Structure of *B. suillus*

Population level genetic data of the Cape Dune mole-rat reveal a diverse (H_E_ = 0.69) population occurring at this site. There is no isolation by distance in either subsample of the population studied. We cannot infer philopatry as noted in other rodents (e.g. [46]) and likely given a short presumed generation time (1–2 years, based on similar species; [47]), although the lack of data from females forestalls a definite conclusion. The population at this study site is not panmictic; there is a low but significant genetic differentiation of the two physically disparate subsamples taken within the population (F_ST_, 0.01, P>0.001). This result is largely expected for a fossorial species with low vagility [48], [49] due to the high costs associated with movement below ground [50]. However the migrant analysis does suggest that bidirectional movement occurs between the two regions subsampled in this study. *B. suillus* are observed moving above-ground and may thus use this energetically cheaper form of locomotion to reduce the costs of dispersing over this 1 km distance. The much smaller naked mole-rat, *Heterocephalus glaber*, has been documented moving over 2 km from its natal burrow when dispersing [48].

### Can we Determine a Limit to Distances Across Which a Sire Will Father Progeny?

Cape dune mole-rats are strictly solitary and highly aggressive to conspecifics within laboratory settings (Bennett and Faulkes 2000). Consequently, the type and frequency of social interactions within and between the sexes remains almost completely unknown. It has been suggested that male *B. suillus* will position burrows adjacent to those of females [51] with longer tunnels increasing the number of females as nearest neighbours and hence improving the chances of successful mate acquisition (sensu [52]). Tunnel lengths have been shown to extend for up to 400 m with as much as 140 m of new tunnel excavated per month [24].

In this study we find a surprisingly low sire assignment rate given known burrow extent and considering the breadth of sampling of potential sires. Females in this study site are located a mean distance of 340 m from one another allowing males to position themselves between multiple female burrow systems. As such we might expect sire assignment distances to be below this inter-female distance due to optimal positioning. The maximum sire-to-progeny distance identified here is 2149 m assuming that all sires were indeed sampled. It is unlikely that a male would have burrow connections to an adjacent burrow system beyond a 500 m straight distance. How male *B. suillus* travel beyond adjacent burrow systems is currently unknown. For some mole-rat species, surface movement is avoided to the extent that even dispersal is known to occur under the ground [53]. Mole-rat vision is rudimentary [54] and thus surface activity would expose individuals to high risks of predation. Despite this, above-ground travel has been invoked for males siring offspring over several hundred metres away in a low density *H. argenteocinereus* population [8]. The combination of the low assignment rate and high upper limit to sire-progeny distances seen in this study supports this suggestion that males of subterranean species may be much less reluctant to travel above-ground than previously thought. Another clue in the data presented is the significantly lower relatedness within the progeny in each region, again implicating paternity from non-resident males. It is likely that above-ground movement is directed through a combination of auditory and olfactory cues. Seismic signals are known to be used for communication in mole-rats [55], in the form of ‘drumming’ with the hind feet. It has been noted that *Georychus capensis* have sexual differences in drumming behaviour [47], and urine is known to convey information regarding sexual condition in other subterranean rodents (e.g. *Ctenomys talarum* [56]).These sensory cues will allow identification of potential mates without entering unfamiliar burrow systems.

### How Common is Multiple Paternity?

Polygyny is typical in mammalian reproduction and is strongly influenced by male behaviour such as competition, spatial organisation, and strategies refined to maximise reproductive success [57]. Despite male attempts to associate spatially with females, solitary females are difficult to monopolise [58]. Multiple paternity within litters is a common feature of polygynous systems and can reveal to some extent the interactions occurring in a cryptic system where behavioural observation is difficult. The real proportion of litters exhibiting multiple sires in the *B. suillus* population investigated here is likely to be somewhere close to the 5/19 predicted by within-litter sibship. Due to the potentially strong influences of genotyping error in allele counts to determine multiple paternity, and the low sire assignment rate seen here, this figure cannot be specified more accurately. Based on the probability of differential gamete inheritance we might allow for a 25% confidence interval around this estimate so potentially a proportion of 0.20-0.33 litters will show multiple paternity.This estimate is higher than the incidence of multiple paternity seen in other solitary mole-rat species (*H. argenteocinereus*, ∼10% [8]; *Spalax ehrenbergi*, ∼0% [21]).

High levels of multiple paternity (up to 80%) have been recorded for rodents with low population viscosity [59]. High levels of multiple paternity might be expected in this study population because of the artificially large size and high density of this population within a predominantly human modified habitat with limited natural predators. High density would increase mating opportunities by bringing opposite sexed individuals into closer physical proximity. Corresponding selection for strong inbreeding avoidance mechanisms might also be expected, as seen in some vole species [60].

The data here suggest that polyandry occurs in this population. Female fitness returns from increasing the number of potential fathers could be those of sperm competition [62]. It is interesting to note that multiple paternity is seen even in socially monogamous mole-rat breeding systems; the common mole-rat (*Cryptomys hottentotus*) is one such system with extra-pair mating seen between colonies [62]. Extra-pair extra-colonial mating has been hypothesised as a mechanism for inbreeding avoidance, characterised by increased within-litter heterozygosity [63]. Extra-pair paternity is also seen in such eusocial systems as the naked mole-rat [64], [65]. In these systems, high relatedness (coefficients of 0.5 or greater) results in a higher fitness trade-off between philopatry and assisting the survival of siblings over the risks of dispersal to form new colonies [66]. We might expect that the reduction in coefficient of relatedness associated with multiple paternity would reduce the fitness benefit of philopatry sufficiently to favour dispersal. For eusocial colonies this implies either a very high cost of dispersal, or that multiple paternity is present only at low levels. Absence of such social restrictions in the solitary species, such as *B. suillus*, would imply that multiple paternity is expected to occur at a much higher level.

### Does Morphological Information or Sex Ratio Help Define Population Dynamics?

It has been noted that male *B. suillus* growth rate declines relative to that of the females at around two to three years of age [67]. High variation in male mass may reflect a period where males lose condition as they either need to search extensively for females or compete with other males for access to females. In some species mass or condition is proposed to determine dispersal distances [46]. Dispersing males are likely to experience higher mortality, successful individuals being those larger males able to establish and defend their burrows resulting in the observed bi-modal size distribution. High male mortality may explain the female biased sex ratio on this site. Sex ratios have the potential to reveal cryptic patterns within the dynamics of mating systems and among different sources of selective pressures. Sex-allocation theory stipulates an even number of each sex unless there are differential costs or fitness returns [68], [69]. Although not significant, a skewed sex ratio can be seen from birth in this population (0.76, n = 51) perhaps suggesting a predisposition of the mating system such as condition dependant sex allocation [70], but more data would be needed to confirm this.

As the first population level genetic study on *B. suillus* the information provided here gives an invaluable insight into this species. It is likely that the high degree of anthropogenic encroachment on organisms such as mole-rats will result in lowered connectivity to other areas. Population isolation has undoubtedly become more commonplace, particularly in low vagility subterranean organisms, and studies of populations such as this one are useful in gauging the result of this isolation. Such insights into the incidence of multiple paternity and polyandry in conjunction with above ground movement described here allow greater understanding of these subterranean organisms. Only through knowledge of these behavioural processes will we be able to describe those selective factors responsible for the generation of the enormous diversity of lifestyles noted in this unusual family of rodents.

## Supporting Information

Appendix S1 Summary information for each locus including number of alleles (A), frequency of null alleles (Null), expected and observed heterozygosity (H_O_, H_E_), and F_IS_ (Probability of significant deviation from Hardy-Weinberg equilibrium; P<0.05 = *, P<0.01 = **, P<0.001 = ***).
(DOCX)

Appendix S2 **Sire assignments for regions 1 (a) and 2 (b) across all models showing sire identity (sire-progeny distances in metres), probability of assignment, and number of mismatches between sire and progeny genotypes (NA denotes no sire assignment made).**
(DOCX)

Appendix S3 **Plots showing the shape and number of either equal (E) or variable (V) volume components; (a) male length, the best BIC values were: V,2 (-6255), V,3 (-6264), E,2 (-6270), and (b) male mass, the best BIC values were:E,2 (-8853.623), V,2(-8858), E,3 (-8866).**
(TIF)
