# Supplementary material for: How Attractive Is the Girl Next Door? An Assessment of Spatial Mate Acquisition and Paternity in the Solitary Cape Dune Mole-Rat, Bathyergus suillus
Source: PLoS One. 2012 Jun 29;7(6):e39866. doi: 10.1371/journal.pone.0039866 (PMC3387204; doi:10.1371/journal.pone.0039866)
Supplement: Appendix S1 — Summary information for each locus including number of alleles (A), frequency of null alleles (Null), expected and observed heterozygosity (HO, HE), and FIS (Probability of significant deviation from Hardy-Weinberg equilibrium; P<0.05 = *, P<0.01 = **, P<0.001 = ***). (DOCX) [file pone.0039866.s001.docx]

| \| \| **Locus** \| **A** \| **Null** \| **H_E_** \| **H_O_** \| **F_IS_** \| \| --- \| --- \| --- \| --- \| --- \| --- \| \| Bsuil06 \| 19 \| 0.09 \| 0.9 \| 0.83 \| 0.08 \| \| \| D5 \| 13 \| 0.17 \| 0.88 \| 0.56 \| 0.36*** \| \| Cmech04 \| 13 \| 0.04 \| 0.87 \| 0.82 \| 0.05 \| \| Bsuil04 \| 14 \| 0.02 \| 0.84 \| 0.83 \| 0.02 \| \| Gcap07 \| 10 \| 0.02 \| 0.84 \| 0.83 \| 0.02 \| \| Ch1 \| 11 \| 0.06 \| 0.8 \| 0.78 \| 0.02 \| \| Gcap10 \| 12 \| 0.04 \| 0.78 \| 0.75 \| 0.04 \| \| Chott03 \| 7 \| 0 \| 0.73 \| 0.76 \| -0.04 \| \| Ch3 \| 5 \| 0.01 \| 0.73 \| 0.76 \| -0.04 \| \| BS07 \| 8 \| 0.05 \| 0.72 \| 0.68 \| 0.06*** \| \| Chott05 \| 7 \| 0.03 \| 0.64 \| 0.64 \| -0.01 \| \| D7 \| 8 \| 0.02 \| 0.63 \| 0.59 \| 0.07 \| \| Gcap02 \| 7 \| 0 \| 0.62 \| 0.63 \| -0.03 \| \| Cmech09 \| 6 \| 0.07 \| 0.62 \| 0.54 \| 0.14* \| \| D1 \| 5 \| 0.32 \| 0.6 \| 0.12 \| 0.80*** \| \| BS01 \| 5 \| 0.01 \| 0.59 \| 0.62 \| -0.05 \| \| Bsuil02 \| 5 \| 0 \| 0.51 \| 0.53 \| -0.05 \| \| Cmech03 \| 10 \| 0.06 \| 0.44 \| 0.43 \| 0.01 \| \| Bsuil01 \| 7 \| 0.01 \| 0.33 \| 0.32 \| 0.01 \| \|  \|  \|  \|  \|  \| \| --- \| --- \| --- \| --- \| --- \| --- \| --- \| --- \| --- \| --- \| --- \| --- \| --- \| --- \| --- \| --- \| --- \| --- \| --- \| --- \| --- \| --- \| --- \| --- \| --- \| --- \| --- \| --- \| --- \| --- \| --- \| --- \| --- \| --- \| --- \| --- \| --- \| --- \| --- \| --- \| --- \| --- \| --- \| --- \| --- \| --- \| --- \| --- \| --- \| --- \| --- \| --- \| --- \| --- \| --- \| --- \| --- \| --- \| --- \| --- \| --- \| --- \| --- \| --- \| --- \| --- \| --- \| --- \| --- \| --- \| --- \| --- \| --- \| --- \| --- \| --- \| --- \| --- \| --- \| --- \| --- \| --- \| --- \| --- \| --- \| --- \| --- \| --- \| --- \| --- \| --- \| --- \| --- \| --- \| --- \| --- \| --- \| --- \| --- \| --- \| --- \| --- \| --- \| --- \| --- \| --- \| --- \| --- \| --- \| --- \| --- \| --- \| --- \| --- \| --- \| --- \| --- \| --- \| --- \| --- \| --- \| --- \| --- \| --- \| --- \| --- \| --- \| \|  \|  \|  \|  \|  \|  \| \|  \|  \|  \|  \|  \|  \| \|  \|  \|  \|  \|  \|  \| \|  \|  \|  \|  \|  \|  \| \|  \|  \|  \|  \|  \|  \| \|  \|  \|  \|  \|  \|  \| \|  \|  \|  \|  \|  \|  \| \|  \|  \|  \|  \|  \|  \| \|  \|  \|  \|  \|  \|  \| \|  \|  \|  \|  \|  \|  \| \|  \|  \|  \|  \|  \|  \| \|  \|  \|  \|  \|  \|  \| \|  \|  \|  \|  \|  \|  \| \|  \|  \|  \|  \|  \|  \| \|  \|  \|  \|  \|  \|  \| \|  \|  \|  \|  \|  \|  \| \|  \|  \|  \|  \|  \|  \| \|  \|  \|  \|  \|  \|  \| |  |  |  |
| --- | --- | --- | --- | --- | --- | --- | --- | --- | --- | --- | --- | --- | --- | --- | --- | --- | --- | --- | --- | --- | --- | --- | --- | --- | --- | --- | --- | --- | --- | --- | --- | --- | --- | --- | --- | --- | --- | --- | --- | --- | --- | --- | --- | --- | --- | --- | --- | --- | --- | --- | --- | --- | --- | --- | --- | --- | --- | --- | --- | --- | --- | --- | --- | --- | --- | --- | --- | --- | --- | --- | --- | --- | --- | --- | --- | --- | --- | --- | --- | --- | --- | --- | --- | --- | --- | --- | --- | --- | --- | --- | --- | --- | --- | --- | --- | --- | --- | --- | --- | --- | --- | --- | --- | --- | --- | --- | --- | --- | --- | --- | --- | --- | --- | --- | --- | --- | --- | --- | --- | --- | --- | --- | --- | --- | --- | --- | --- | --- | --- | --- | --- | --- | --- | --- | --- | --- | --- | --- | --- | --- | --- | --- | --- | --- | --- | --- | --- | --- | --- | --- | --- | --- | --- | --- | --- | --- | --- | --- | --- | --- | --- | --- | --- | --- | --- | --- | --- | --- | --- | --- | --- | --- | --- | --- | --- | --- | --- | --- | --- | --- | --- | --- | --- | --- | --- | --- | --- | --- | --- | --- | --- | --- | --- | --- | --- | --- | --- | --- | --- | --- | --- | --- | --- | --- | --- | --- | --- | --- | --- | --- | --- | --- | --- | --- | --- | --- | --- | --- | --- | --- | --- | --- | --- | --- | --- | --- | --- | --- | --- | --- | --- | --- | --- | --- | --- | --- | --- | --- |
