# Supplementary material for: How Attractive Is the Girl Next Door? An Assessment of Spatial Mate Acquisition and Paternity in the Solitary Cape Dune Mole-Rat, Bathyergus suillus
Source: PLoS One. 2012 Jun 29;7(6):e39866. doi: 10.1371/journal.pone.0039866 (PMC3387204; doi:10.1371/journal.pone.0039866)
Supplement: Appendix S2 — Sire assignments for regions 1 (a) and 2 (b) across all models showing sire identity (sire-progeny distances in metres), probability of assignment, and number of mismatches between sire and progeny genotypes (NA denotes no sire assignment made). (DOCX) [file pone.0039866.s002.docx]

| (a) |  | No spatial data – Categorical allocation | | | |  |  | Spatial data – Full probability | | |  |  |
| --- | --- | --- | --- | --- | --- | --- | --- | --- | --- | --- | --- | --- |
|  | ALLsire |  |  | Ussire |  |  | ALLsire |  |  | Ussire |  |  |
| Progeny | Sire | Probability | mismatches | Sire | Probability | mismatches | Sire | Probability | mismatches | Sire | Probability | mismatches |
| 1.1.1 | NA | - | - | NA | - | - | NA | - | - | NA | - | - |
| 1.1.2 | NA | - | - | NA | - | - | NA | - | - | NA | - | - |
| 1.2.1 | NA | - | - | NA | - | - | NA | - | - | NA | - | - |
| 1.2.2 | NA | - | - | NA | - | - | NA | - | - | NA | - | - |
| 1.2.3 | 274(192) | 0.98 | 2 | NA | - | - | NA | - | - | NA | - | - |
| 1.3.1 | 227(316) | 0.52 | 2 | NA | - | - | NA | - | - | NA | - | - |
| 1.3.2 | NA | - | - | NA | - | - | NA | - | - | NA | - | - |
| 1.4.1 | NA | - | - | NA | - | - | NA | - | - | NA | - | - |
| 1.4.2 | 1260(34) | 1 | 0 | 1260 | 1 | 0 | 1260(34) | 1 | 0 | 1260 | 1 | 0 |
| 1.4.3 | 1260(34) | 0.99 | 0 | 1260 | 0.94 | 0 | 1260(34) | 1 | 0 | 1260 | 1 | 0 |
| 1.5.1 | 715(732) | 0.92 | 2 | NA | - | - | NA | - | - | NA | - | - |
| 1.5.2 | NA | - | - | NA | - | - | NA | - | - | NA | - | - |
| 1.5.3 | NA | - | - | NA | - | - | NA | - | - | NA | - | - |
| 1.6.1 | 97(1977) | 0.46 | 2 | NA | - | - | NA | - | - | NA | - | - |
| 1.6.2 | 231(1850) | 0.98 | 2 | NA | - | - | NA |  |  | NA | - | - |
| 1.6.3 | NA | - | - | NA | - | - | NA | - | - | NA | - | - |
| 1.7.1 | NA | - | - | NA | - | - | NA | - | - | NA | - | - |
| 1.7.2 | NA | - | - | NA | - | - | NA | - | - | NA | - | - |
| 1.8.1 | NA | - | - | NA | - | - | NA | - | - | NA | - | - |
| 1.8.2 | NA | - | - | NA | - | - | NA | - | - | NA | - | - |
| 1.9.1 | 1042(411) | 0.91 | 2 | NA | - | - | 1042(411) | 1 | 2 | 1042 | 2 | 0.58 |
| 1.9.2 | 1042(411) | 0.89 | 2 | NA | - | - | 1042(411) | 1 | 2 | 1042 | 2 | 0.51 |
| 1.9.3 | 1042(411) | 0.89 | 2 | NA | - | - | 1042(411) | 1 | 2 | 1042 | 2 | 0.51 |

| (b) |  | No spatial data – Categorical allocation | | | |  |  | Spatial data – Full probability | | |  |  |
| --- | --- | --- | --- | --- | --- | --- | --- | --- | --- | --- | --- | --- |
|  | ALLsire |  |  | Ussire |  |  | ALLsire |  |  | Ussire |  |  |
| Progeny | Sire | Probability | mismatches | Sire | Probability | mismatches | Sire | Probability | mismatches | Sire | Probability | mismatches |
| 2.1.1 | NA | - | - | NA | - | - | NA | - | - | NA | - | - |
| 2.1.2 | 1114(1969) | 0.58 | 2 | NA | - | - | NA | - | - | NA | - | - |
| 2.2.1 | 111(159) | 0.41 | 2 | NA | - | - | 1256(795) | 0.4 | 2 | NA | - | - |
| 2.3.1 | NA | - | - | NA | - | - | NA | - | - | NA | - | - |
| 2.3.2 | NA | - | - | NA | - | - | 174(260) | 0.59 | 2 | NA | - | - |
| 2.3.3 | 600(1426) | 0.99 | 2 | NA | - | - | 1168(323) | 0.49 | 2 | NA | - | - |
| 2.3.4 | NA | - | - | NA | - | - | NA | - | - | NA | - | - |
| 2.4.1 | NA | - | - | NA | - | - | NA | - | - | NA | - | - |
| 2.4.2 | NA | - | - | NA | - | - | NA | - | - | NA | - | - |
| 2.5.1 | 787(567) | 0.25 | 1 | 787(567) | 0.23 | 1 | 787(567) | 0.5 | 1 | NA | - | - |
| 2.5.2 | 135(306) | 0.96 | 2 | NA | - | - | 135(306) | 0.6 | 2 | NA | - | - |
| 2.6.1 | NA | - | - | NA | - | - | 1387(250) | 0.8 | 2 | NA | - | - |
| 2.6.2 | NA | - | - | NA | - | - | 170(276) | 0.47 | 2 | NA | - | - |
| 2.6.3 | 1231(2149) | 1 | 2 | NA | - | - | 1231(2149) | 1 | 2 | NA | - | - |
