# Supplementary figures and images for: How Attractive Is the Girl Next Door? An Assessment of Spatial Mate Acquisition and Paternity in the Solitary Cape Dune Mole-Rat, Bathyergus suillus
Source: PLoS One. 2012 Jun 29;7(6):e39866. doi: 10.1371/journal.pone.0039866 (PMC3387204; doi:10.1371/journal.pone.0039866)

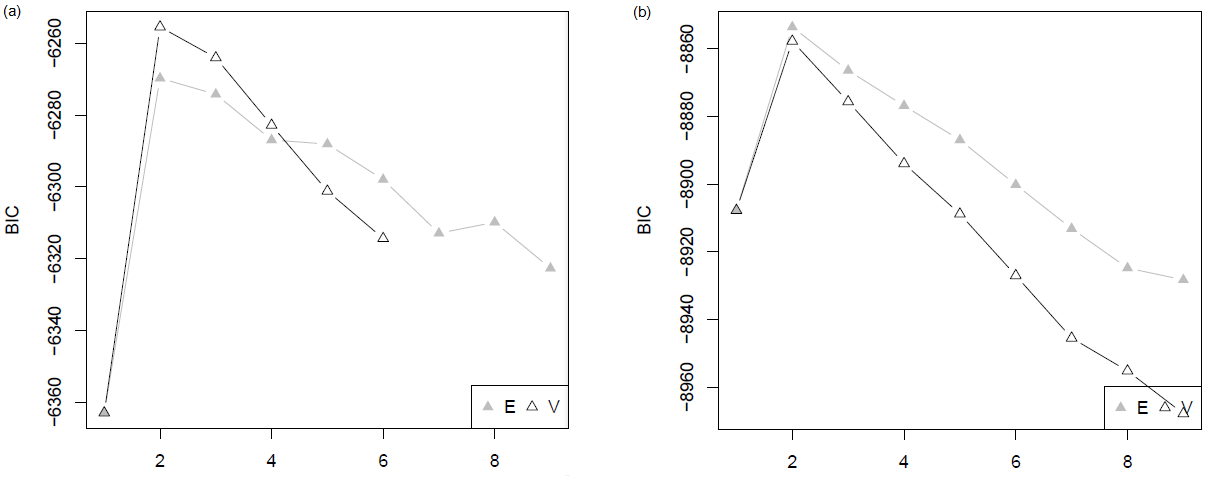

Supplement: Appendix S3 — Plots showing the shape and number of either equal (E) or variable (V) volume components; (a) male length, the best BIC values were: V,2 (-6255), V,3 (-6264), E,2 (-6270), and (b) male mass, the best BIC values were:E,2 (-8853.623), V,2(-8858), E,3 (-8866). (TIF) [file pone.0039866.s003.tif]
